# Effects of eccentric loading on performance of concrete columns reinforced with glass fiber-reinforced polymer bars

**DOI:** 10.1038/s41598-023-47609-4

**Published:** 2024-01-22

**Authors:** Nasim Shakouri Mahmoudabadi, Alireza Bahrami, Saba Saghir, Afaq Ahmad, Muhammad Iqbal, Mohamed Elchalakani, Yasin Onuralp Özkılıç

**Affiliations:** 1 Department of Civil Engineering, The University of Memphis, Memphis, TN 38152 USA; 2 Department of Building Engineering, Energy Systems and Sustainability Science, Faculty of Engineering and Sustainable Development, University of Gävle, 801 76 Gävle, Sweden; 3 National Highway Authority, Islamabad, Pakistan; 4 Department of Mechanical Engineering, CECOS University of IT and Emerging Sciences, Peshawar, Pakistan; 5 Department of Civil Engineering, The University of Western Australia, Perth, Australia; 6 Department of Civil Engineering, Faculty of Engineering, Necmettin Erbakan University, 42100 Konya, Turkey; 7 Department of Civil Engineering, Lebanese American University, Byblos, Lebanon

**Keywords:** Civil engineering, Mechanical engineering

## Abstract

Glass fiber-reinforced polymer (GFRP) reinforcements are superior to traditional steel bars in concrete structures, particularly in vertical elements like columns, and offer significant advantages over conventional steel bars when subjected to axial and eccentric loadings. However, there is limited experimental and numerical research on the behavior of GFRP-reinforced concrete (RC) columns under eccentric loading having different spacing of stirrups. In this study, six specimens were cast under three different values of eccentricities (25 mm, 50 mm, and 75 mm) with two groups of stirrups spacing (50 mm and 100 mm). The experimental results showed that by increasing the eccentricity value, there was a reduction in the load-carrying capacity of the specimens. The finite element ABAQUS software was used for the numerical investigation of this study. The results from the finite element analysis (FEA) were close to the experimental results and within the acceptable range. The maximum difference between the experimental and FEA results was 3.61% for the axial load and 12.06% for the deformation.

## Introduction

Glass fiber-reinforced polymers (GFRPs) are extensively used as an innovative reinforcement material, replacing traditional steel reinforcements in reinforced concrete (RC) constructions. This is primarily thanks to their corrosion-free properties^1^. According to Ephraim et al.^2^, 40% fiber in GFRPs resulted in 25% more ductility compared to the recommendations in ACI 440^3^. Jabbar and Farid^4^ also noted that GFRP bars not only provide higher corrosion resistance but also offer the tensile strength 13% greater and the tensile yield strain 58% higher than steel. In the last two decades, numerous studies have emphasized the application of GFRPs in axial members with solid cross-sections, investigating various loading circumstances. The cover spalling was evaluated by Tobbi et al.^5^ who found that the application of GFRP spiral reinforcement increased the columns' strength and ductility, providing lateral confinement. The order of columns, from lowest to highest ultimate axial load-carrying capacity, was rectangular, square, and circular columns, as stated by Raval and Dave^6^. However, significant enhancement in the axial strength and corresponding deviation was achieved by applying fiber-reinforced polymer (FRP) wrapping to RC circular columns^7^. Columns reinforced with FRP bars demonstrated considerable compressive strengths ranging from 10% to 86% and modulus of elasticity values ranging from 65% to 97%, depending on the type of the fibers. These percentages reflected the enhanced performance compared to traditional RC columns without FRP reinforcements^8^. Unlike steel-RC columns, GFRP-RC columns experience failure when both concrete and GFRP bars are crushed simultaneously, according to Gamal^9^. In a different context, Khodadadi et al.^10^ utilized a deep neural network model for a parametric study on the compressive strength of FRP-confined concrete columns. The assessment of relationships between input variables and ANN model outputs, along with examining interaction effects between pairs of input variables, revealed that the confined compressive strength indicated minimal sensitivity to the elastic modulus of composite layers with thicknesses of 2 mm or less. This study illustrated how advanced computational methods can help understand the behavior of FRP-confined concrete columns and offer valuable insights into their structural performance.

Furthermore, Shaaban^11^ delved into the behavior of eccentrically loaded concrete-filled steel box columns to enhance their structural performance. This investigation included comparing plain concrete and steel fiber-RC. It was found that including 1–4% steel fibers remarkably improved the performance of concrete-filled steel box columns when subjected to eccentric loads. Additionally, Shaheen et al.^12^ conducted experimental research on rectangular RC columns strengthened with FRP wraps subjected to cyclic lateral and axial loadings. Their study evaluated the behavior of these retrofitted columns and provided valuable recommendations for utilizing FRP to improve the seismic performance of rectangular columns. These studies underscore the importance of innovative materials and retrofitting techniques in optimizing the structural response of various column types under different loading conditions. It is not well known how GFRP bars would behave in reinforced columns under compressive loading conditions, contrary to the existing understanding of GFRP bars under tension. As a result, many standards, such as ACI 440^3^, restrict designers from utilizing GFRP bars in compression members, although CSAS6-06^13 ^has not discussed this compressive behavior of GFRP bars. Limited research works have been done on the use of eccentrically loaded columns reinforced with FRP ^14–28^. Furthermore, few studies have assessed the behavior of GFRP bars embedded in concrete columns under pure axial load, but minimal experimental and numerical data exist on the response of longitudinal and transverse GFRP-RC columns under eccentric, concentric, and flexural loadings. Steel reinforcing used in bridge piers is prone to corrosion due to exposure to chloride attack from flowing water^14–21^. To study the serviceability of GFRP bars, the number of field tests performed and GFRP bars exposed to harsh environments did not show any signs of degradation during 6–8 years^20^. The constructability issues limit the use of GFRP bars because of their brittle nature as opposed to ordinary steel bars already being utilized in the RC columns^26^. Necessary investigation on the induction of GFRP bars as replacement of steel compression reinforcement is needed to understand the behavior of GFRP-RC columns under eccentric loading.

An examination was conducted to analyze the performance of GFRP-RC columns. The GFRP column's response was discovered to closely resemble that of a conventional RC column with 1% reinforcement ratio of the cross-section, as evidenced by previous studies^29–34^. The load-carrying capacity of a GFRP-RC column for axial loads, subjected to the eccentric loading having different reinforcement ratios, was considerably affected and reduced in comparison to the concrete column made of steel reinforcement. It was found that the GFRP-RC columns subjected to eccentric loading experienced considerable lateral displacements, leading to a decrease in the axial load-carrying capacity. According to an experimental study on GFRP-RC square columns under axial stress, GFRP-RC specimens had lower load-carrying capacity than steel-RC specimens. Furthermore, steel-RC specimens outperformed GFRP-RC specimens in terms of the ductility under concentric compressive stress. However, in this experiment, steel stirrups were employed as a kind of confinement^29^. Steel corrosive qualities may affect the key attributes such as the strength and ductility of steel bars in RC structures. The use of an alternative material for steel stirrups is important to consider. Stirrups are extremely vulnerable to severe environmental impacts due to their smaller diameter and less concrete cover compared to the longitudinal bars^35^.

Utilizing the finite element method (FEM) offers a cost-effective and time-efficient approach to analyze FRP composites, reducing the need for experimental setups^36–38^. In a comparative analysis performed by Havlasek^39^, the concrete damage plasticity model was assessed against experimental data. Circular concrete-filled steel tube columns exhibited superior axial performance compared to their square and rectangular counterparts, according to Bahrami et al.^40^. He et al.^41^ did the finite element analysis (FEA) on hollow composite tubular columns reinforced with GFRP bars, observing a greater capacity to transport loads through either reducing the hollow ratio or enhancing the concrete strength. Rashid and Bahrami^42^ evaluated how well thin-walled composite steel–concrete columns filled with FRP and carbon fiber-reinforced polymer function structurally. Developing analytical studies using finite element models validated by experimental results to investigate the effects of axial loading on structural elements is not limited to critical structural elements. For instance, Nateghi^43^ analytically examined the behavior of finite element models of multilayer accordion metallic dampers under axial cyclic loading, providing insights into diverse structural enhancement strategies. The results demonstrated the versatility of FEM in assessing various structural components and systems, offering a valuable tool for understanding their behavior under different loading conditions and optimizing their performance.

Based on an extensive literature review, it was determined that numerical investigation is necessary to understand the axial response of GFRP-RC columns when subjected to eccentric loading. Therefore, this study has focused on employing the ABAQUS software for FEA to analyze the axial response of GFRP-RC columns. The significance of this research lies in its ability to elucidate the axial response of GFRP-RC columns in the presence of eccentric loading and pave the way for future parametric studies, eliminating the necessity for laborious, time-intensive, and experimental destructive testings. In a related context, Namadchi et al.^44^ introduced an innovative time integration method to improve the accuracy and efficiency of solving structural dynamic problems by damping out the spurious oscillations of the high-frequency modes. However, some studies underscore the significance of advanced numerical methods across materials and structural analysis domains. In addition, Najmi and Hu^45^ used molecular dynamic simulations to examine factors like carbon nanotubes' chirality, length, and hydrogen bonding, impacting the thermal and electrical conductivity of modified polymer composites. This research deepened our understanding of these composites at the molecular level, shedding light on their potential applications.

In light of limited prior research on the behavior of GFRP-RC columns subjected to eccentric loading with varying stirrups spacing, Section "experimental program" of the current article describes the preparation of six column specimens designated as 50-E25, 50-E50, 50-E75, 100-E25, 100-E50, and 100-E75. The first number, E, and the last number in the specimens' designations represent the spacing of stirrups in mm, the eccentric loading, and the value of eccentricity in mm, respectively. The experimental findings emphasized a noticeable reduction in the load-carrying capacity with increasing the eccentricity values. To complement these experimental results, FEA of the columns utilizing ABAQUS was done. Section "FEA" presents the finite element modeling and its specifications. Section "validation of modeling" explains the validation process of modeling. Section "comparison of experimental and numerical results" discusses the obtained comparative results from the experiments and FEA.

## Experimental program

### Materials and their characteristics

#### Concrete

To prepare the column specimens, a standard normal-strength concrete was used, following the guidelines of ASTM/C143^46^. Concrete had a slump of 105 mm and utilized aggregates with a maximum particle size of 10 mm (referring to the maximum diameter of aggregates). The concrete cylinders' mean compressive strength at 28 days was measured 35 MPa with a standard deviation of 3.24 MPa. In order to determine the columns' compressive strength, six cylinders with the dimensions of 0.2 m in height and 0.1 m in diameter were cast, adhering to the specifications of ASTM/C3. These cylinders were then tested in accordance with ASTM/C39^47^ and CSA/S806^48^.

#### Reinforcements

**Figure 1:**
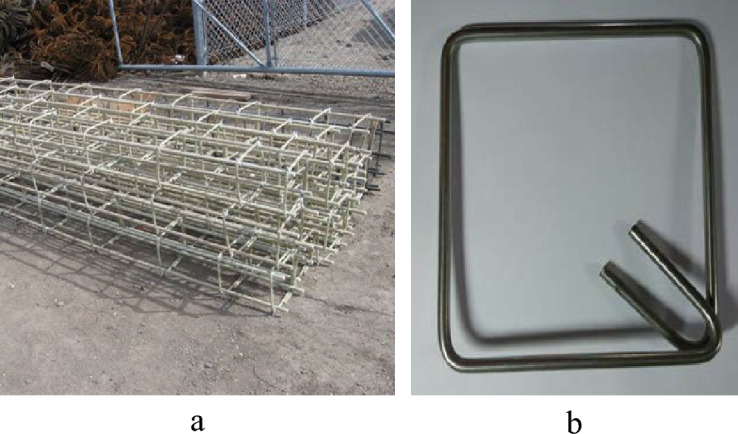
(**a**) GFRP longitudinal bars and cage, (**b**) stainless-steel stirrups.

**Figure 2:**
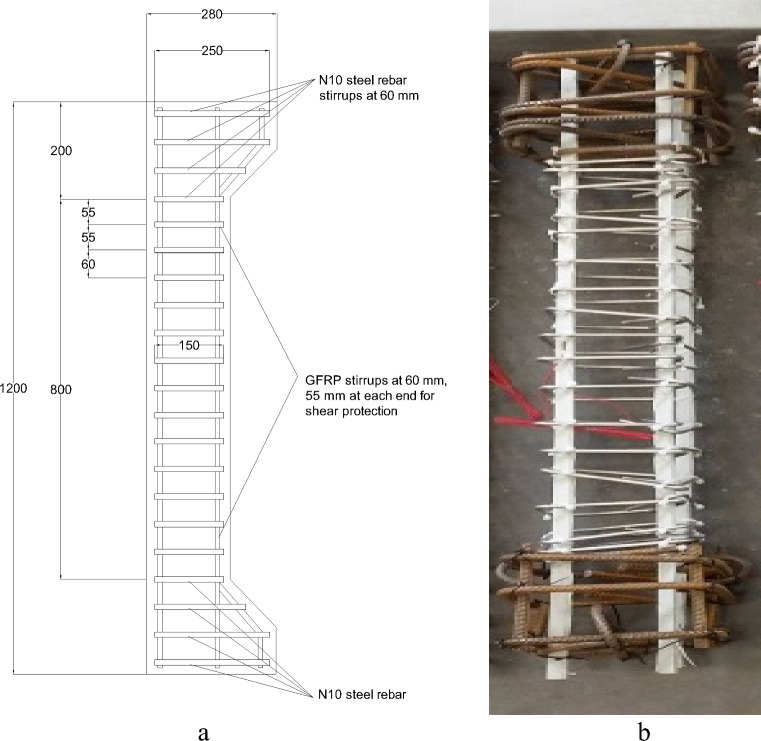
Column specimens: (**a**) details (dimensions are in mm), (**b**) reinforcements in experimental specimen.

The main reinforcements of the GFRP-RC columns were provided by four bars of No. 12 (N12) GFRP (each 11 mm diameter) with 1.17% longitudinal steel ratio. For the eccentric loading, additional steel bars of N12 were applied at the top and bottom of the specimens with the lateral confinement of No. 10 (N10) steel bars at 50 mm, as illustrated in Fig. 1. For the transverse confinement in the middle region, stainless-steel stirrups of No. 6 (N6) @ 50 mm or 100 mm (each 6 mm diameter) as lateral reinforcements were provided (Figs. 1 and 2). Table 1 summarizes the physical and mechanical characteristics of reinforcements.

**Table 1:** Specifications of reinforcements.

Reinforcement type	Nominal diameter (mm)	Area (mm^2^)	Tensile strength (MPa)	Elastic modulus (GPa)
N12 GFRP	11.8	95	750	62.5
N12 steel	11.8	78.54	500	300
N6 stainless-steel	6	28.27	680	310

### Preparation of specimens

**Figure 3:**
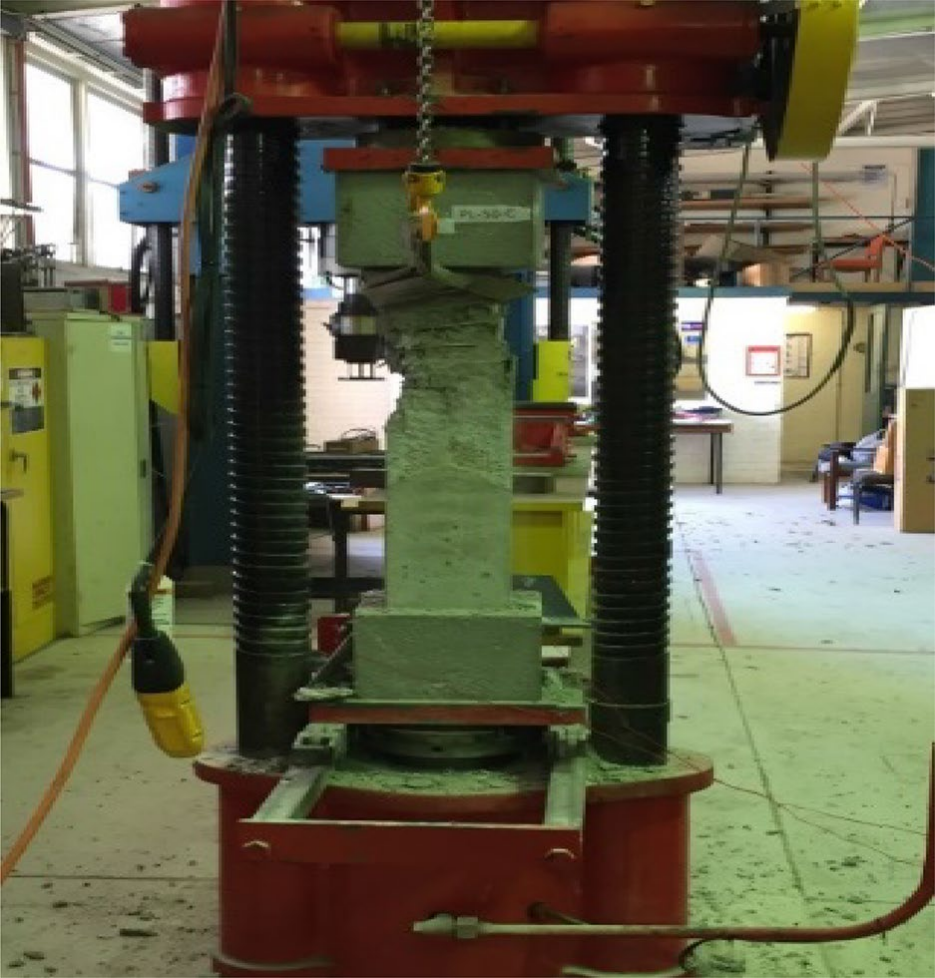
Experimental setup.

Six concrete column specimens were constructed (each column having a height of 1200 mm and a width of 180 mm) with different stirrups spacing and investigated under different eccentric loadings, as listed in Table 2. The last column of Table 2 presents the designations of these six specimens. The first number, E, and the last number in the specimens' designations represent the spacing of stirrups in mm, the eccentric loading, and the value of eccentricity in mm, respectively. Figure 3 depicts the experimental setup.

**Table 2:** Features of tested column specimens.

No.	Spacing of stirrups (mm)	Eccentricity (mm)	Designation
1	50	25	50-E25
2	50	50	50-E50
3	50	75	50-E75
4	100	25	100-E25
5	100	50	100-E50
6	100	75	100-E75

### Test setup and instrumentation

The column specimens were subjected to a 2000 kN monotonic concentric load, applied with a hydraulic cylinder at a pace of 1.5 mm/min to assess their performance. To ensure that failure occurs at the intended location, additional hunches were incorporated at the top and bottom of the specimens, as shown in Fig. 3. A 2000 kN load cell was used to measure the axial load, and a string pot was utilized to monitor the deformation. A system 5000 data logger was employed to continuously record the applied load, axial deformation, and strain during the testing process. As the specimens were loaded, extra attention was paid to the cracking pattern.

### Experimental results

**Figure 4:**
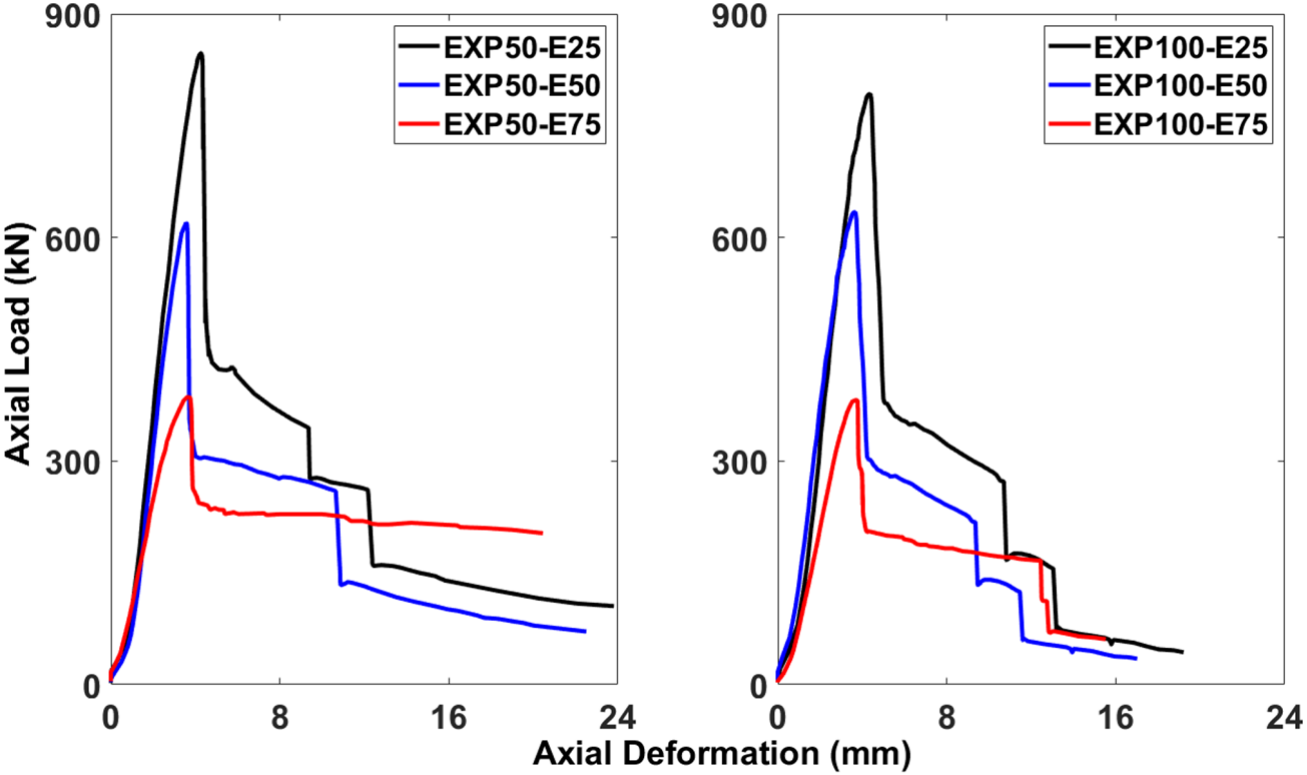
Effect of eccentricity against spacing of stirrups on axial load-carrying capacity of columns.

**Figure 5:**
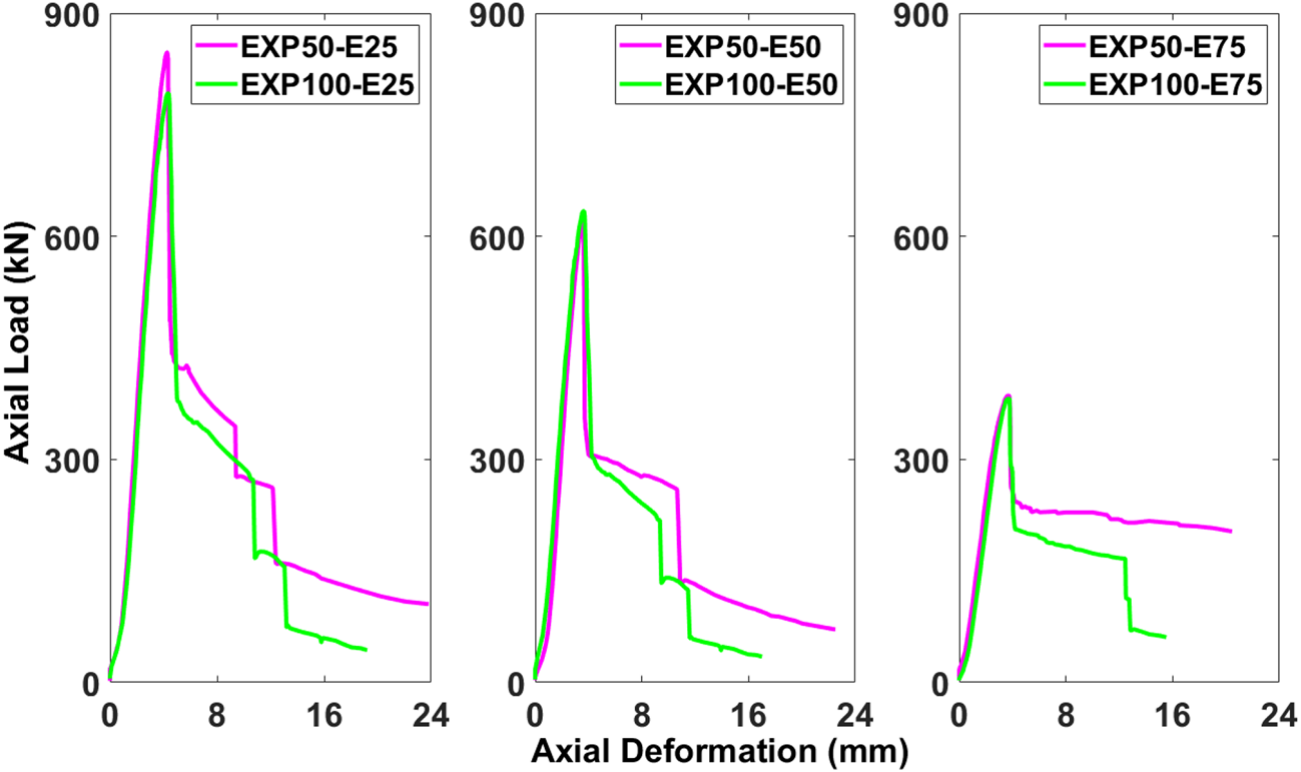
Effect of spacing of stirrups against eccentricity on axial load-carrying capacity of columns.

Figures 4 and 5 display the responses of these six columns in two groups as the effects of different eccentricity values against the same stirrups spacing and the effects of different stirrups spacing against the same eccentricity value, respectively. Also, Table 3 represents the comparison of the experimental values of the ultimate axial load-carrying capacity and deformation for different stirrups spacing and eccentricity values. The specimens having the stirrups spacing of 50 mm against different values of the eccentric loading exhibited the decreased pattern of the load-carrying capacity, i.e., 50-E25 (847.53 kN), 50-E50 (619.43 kN), and 50-E75 (385.64 kN). The reduction in the load-carrying capacity between 50-E25 and 50-E50 was 26.92%, while it was 54.42% between 50-E25 and 50-E70. The same pattern could be observed for the specimens having the stirrups spacing of 100 mm against different values of the eccentric loading, i.e., 100-E25 (792.12 kN), 100-E50 (633.70 kN), and 100-E75 (381.91 kN). The load-carrying capacity was reduced 20% between 100-E25 and 100-E50, however, it was 51.78% between 100-E25 and 100-E70.

The reduction in the load-carrying capacity between 50-E25 and 100-E25 was 6.54%, while it was 0.97% between 50-E75 and 100-E75 (Fig. 5). However, there was an increase in the load-carrying capacity between 50-E50 and 100-E50 as 2.3% (Fig. 4).

**Table 3:** Comparison of experimental values of ultimate axial load-carrying capacity and deformation for different stirrups spacing and eccentricity values.

Specimen	Ultimate axial load-carrying capacity		Deformation	
	Value (kN)	Difference (%)	Value (mm)	Difference (%)
50-E25	847.53	6.54	4.26	− 0.94
100-E25	792.12		4.3	
50-E50	619.43	− 2.3	3.56	− 0.84
100-E50	633.7		3.59	
50-E75	385.64	0.97	3.6	− 3.33
100-E75	381.91		3.72	

## FEA

### Overview

FEA of the RC columns involved creating models for the constituent materials, including concrete, GFRP, and steel reinforcements, and their respective behaviors. FEA of the RC columns was carried out using ABAQUS^49^. Concrete was represented as a 3D solid stress section, while reinforcements were simulated using wire elements with 3D deformability. For ensuring an evenly and gradually distributed load application, appropriate boundary loading and conditions were used to apply to the model. Specifically, fixed boundary conditions were considered on the bottom face of the concrete section to represent the column's connection to the foundation. These conditions included constraining translational and rotational degrees of freedom, preventing rigid body motions. Additionally, a vertically distributed load was applied evenly across the top surface of the concrete section to simulate the axial loading. The validated model was then adjusted for various parameters, including the shape factor of concrete, viscosity parameter, dilation angle, mesh element types, and mesh sizes. Subsequently, an additional parametric study was undertaken by applying the adjusted finite element model to explore additional parameters.

### Modeling concrete

#### Modeling concrete plasticity

**Figure 6:**
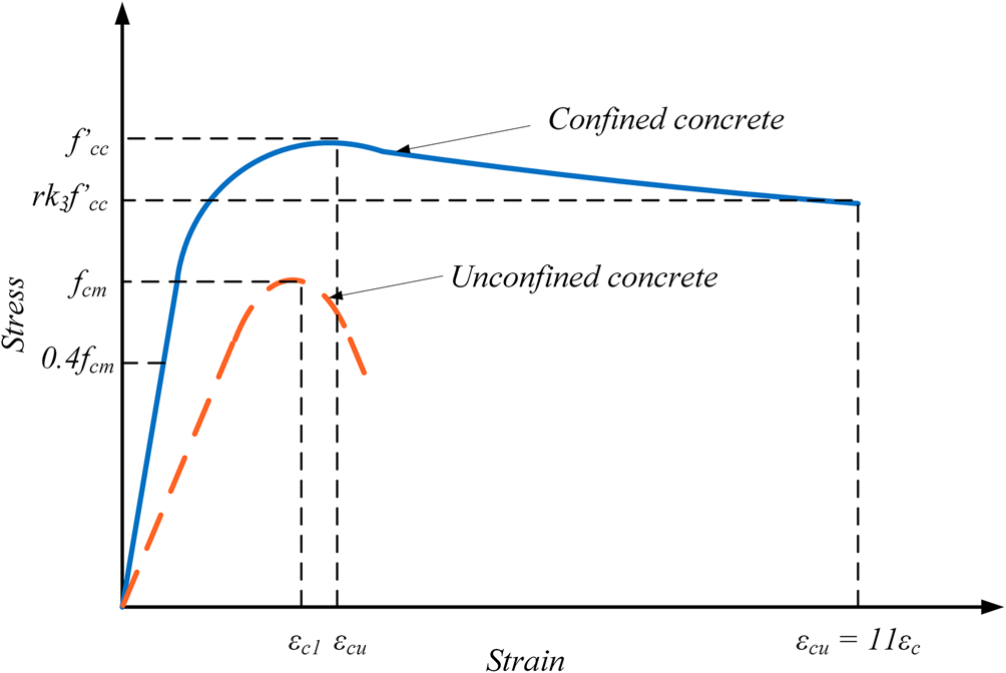
CDP model^50^.

**Figure 7:**
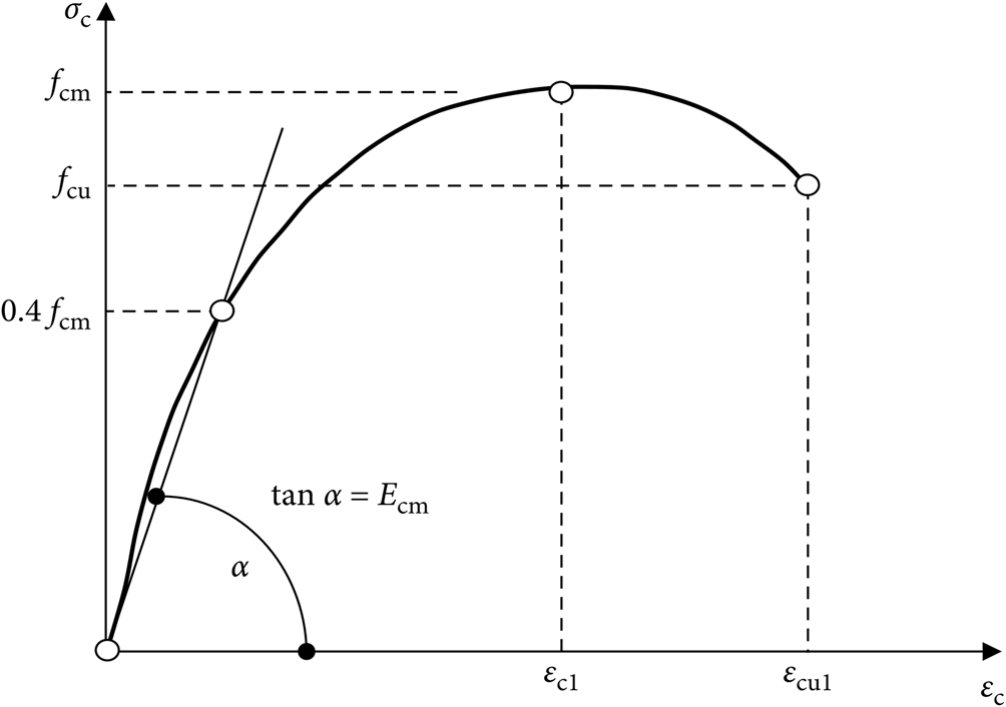
Stress–strain relation of concrete^49^.

**Figure 8:**
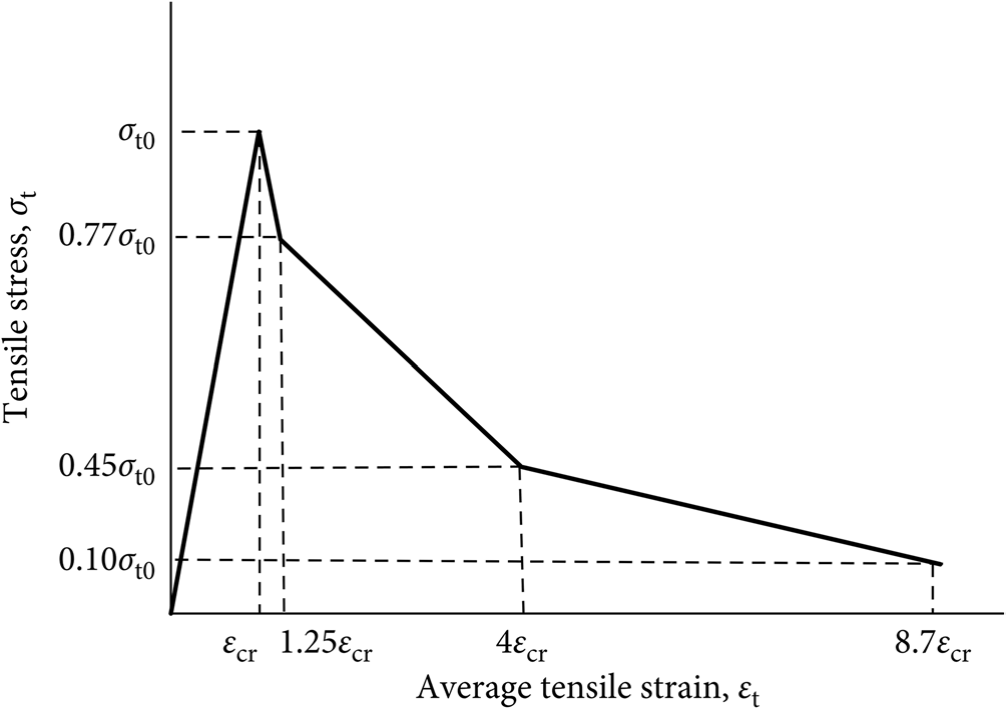
Model for tension stiffening^49^.

Concrete's inelastic response can be described using one of three different models provided by ABAQUS as concrete damage plasticity (CDP), concrete smeared cracking (CSC), and brittle cracking concrete (BCC)^49^. The CDP model, which is based on the plasticity, shares similarities with the other two models in terms of explaining the behavior and failure patterns of concrete, including both the tensile cracking and compressive crushing. However, the CDP model stands out as the most accurate one, providing more precise output results, particularly when used in conjunction with the CSC model (Figs. 6, 7, 8). The maximum stress experienced by confined concrete is denoted as *f'*_*cc*_, while confined concrete failure stress is represented as *rk*_*3*_*f'*_*cc*_, and the corresponding strain is ${\varepsilon }_{cu}$. Similarly, the maximum stress experienced by unconfined concrete is *f*_*cm*_, and the respective strain is ${\varepsilon }_{c1}$.

### Computation of tensile and compressive stresses

By utilizing the uniaxial stress–strain relationship, one can effectively derive the stress versus plastic strain relationship in ABAQUS. This is achieved through the provision of the stress versus inelastic strain data, allowing for the automatic conversion and calculation. Consequently, ${{\varepsilon }_{c}}^{pl}$ and ${{\varepsilon }_{t}}^{pl}$ indicate the corresponding plastic strains in the compression and tension, respectively, while ${{\varepsilon }_{c}}^{.pl}$ and ${{\varepsilon }_{t}}^{.pl}$ represent the rates of the equivalent plastic strains in the compression and tension, respectively. Incorporating variables such as *θ* (temperature) and *f*_*i*_ (another defined variable), as demonstrated in Figs. 6, 7, and 8, the following relationships can be established:

1 $${\sigma }_{t}={\sigma }_{t}({{\varepsilon }_{t}}^{pl},{{\varepsilon }_{t}}^{.pl},\theta ,{f}_{i})$$

2 $${\sigma }_{c}={\sigma }_{c}({{\varepsilon }_{c}}^{pl},{{\varepsilon }_{c}}^{.pl},\theta ,{f}_{i})$$

In the phase of strain softening, when concrete undergoes unloading, its elastic stiffness experiences a decrease. The extent of this decrease in the elastic stiffness depends on factors such as temperature, plastic strains, and other field variables. To characterize this decrease, two damage variables, *d*_*c*_ and *d*_*t*_, are employed, varying between 0 (indicating undamaged material) and 1 (representing fully damaged material), as described in the following manner:

3 $${{d_t = d_t(\varepsilon }_{t}}^{pl},\theta ,{f}_{i})$$

4 $${d_c = d_c{(\varepsilon }_{c}}^{pl},\theta ,{f}_{i})$$

where 0 ≤ *d*_*t*_ and *d*_*c*_ ≤ 1.

The equations below present the stress–strain curves for both uniaxial compressive and tensile loads, with *E*_0_ representing the undamaged concrete's initial elastic stiffness.

5 $${\sigma }_{t}={\left(1-{d}_{t}\right) E}_{0}({\varepsilon }_{t}-{{\varepsilon }_{t}}^{pl})$$

6 $${\sigma }_{c}={\left(1-{d}_{c}\right) E}_{0}({\varepsilon }_{c}-{{\varepsilon }_{c}}^{pl})$$

### Modeling

#### Materials and geometric properties

**Figure 9:**
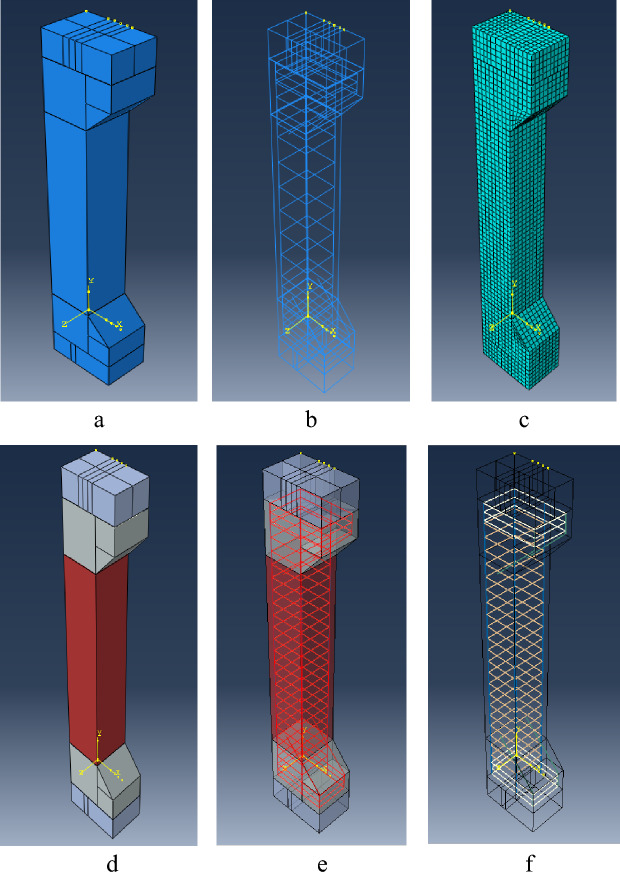
Modeling steps: (**a**) geometry, (**b**) rebars cage, (**c**) meshing, (**d**) assembly of different materials for concrete and steel plates, (**e**,**f**) illustrated materials for GFRP and stainless steel.

A 3D solid feature was utilized to model the concrete core, employing the third dimension as an extrusion method. On the other hand, GFRP and steel bars were represented with a planar third dimension based on a 3D wire frame. Stainless steel was also indicated as a 3D wire frame, which had a translational spacing of either 50 mm or 100 mm and was wrapped around the bars. Due to the superior corrosion resistance of GFRP, a relatively smaller clear cover was used for GFRP reinforcements in accordance with the suggestions^36–38^. In addition, certain additional components of the testing apparatus, like bottom and top steel plates, were simulated as 3D solid features, utilizing standard steel properties. These details can be observed in Fig. 9.

**Figure 10:**
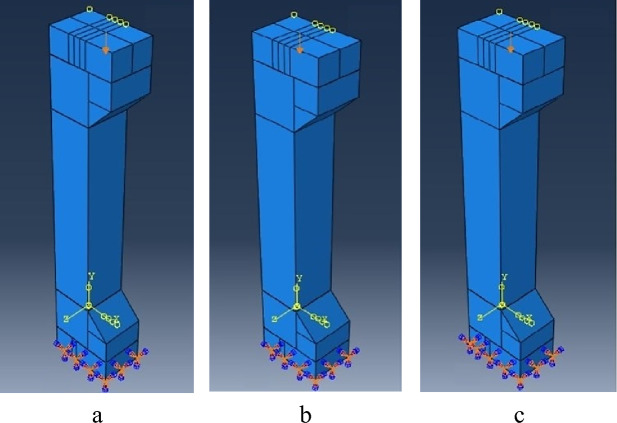
Specimen: (**a**) eccentricity 25 mm, (**b**) eccentricity 50 mm, (**c**) eccentricity 75 mm.

The eccentricities of 25 mm, 50 mm, and 75 mm are shown in Fig. 10. In terms of the flexibility, all components were chosen to be deformable, allowing for deformation under loading and facilitating the measurement of their responses. Due to the relative advantages of the CDP model, it was used for modeling the plastic behavior of concrete. GFRP and steel reinforcements were modeled as linear elastic materials. Table 4 provides input values for FEA by ABAQUS, which have been considered by the authors in this research^36–38^.

**Table 4:** Input values for FEA by ABAQUS.

Parameters	Values	Unit
Increment size of initial loading	0.01	kN
Maximum increment size of loading	0.1	kN
Minimum increment size of loading	1e^-15^	kN
Number of increments	10,000	–

#### Finite element mesh

When it comes to meshing, two important factors to consider are the size of the mesh and mesh element type. In ABAQUS, there are two main sets of the mesh elements available as 3D stress elements and 3D wire elements. The 3D wire elements include sub-types such as T3D2H and T3D3H. On the other hand, the 3D stress elements encompass various types, such as tetrahedral (C3D10H, C3D4H), triangular (C3D15H, C3D6H), and hexahedral (C3D20R, C3D8R) elements. In this particular model, T3D2 elements were utilized to mesh GFRP and steel reinforcements, and they are associated with linear shape functions suitable for truss elements. To simulate concrete, C3D8R elements were used, which are 3D eight-node hexahedral elements employing reduced integration and are associated with appropriate shape functions for hexahedral elements. Ahmad et al.^38^ also validated the appropriateness of utilizing these elements for the static and dynamic analyses of nonlinear systems. Once the type of mesh was defined, validation led to a 20 mm mesh size selection to maintain a close correlation with the experimental results.

#### Interactions and constraints

To establish the interplay between different elements, a tie constraint was employed, using the notion of slave and master surfaces. In the context of a FEA software like ABAQUS, a tie constraint refers to a mathematical formulation that enforces a connection between surfaces or components within the model. Every pair of matching surfaces consisted of a master surface responsible for load transfer and a slave surface as the counterpart. This allowed for the accurate simulation of smooth load transmission from the top steel plate to the concrete column and, ultimately, to the steel plate at the bottom. The embedded region constraint was utilized to simulate the connection between concrete, reinforcement bars, and stirrups, where the call for reinforcements came from embedded elements and concrete functioned as host region. The use of tie constraints is a fundamental technique in FEA, ensuring that different parts of the model interact correctly.

The lower ends of the specimens were securely fixed employing the encased condition. However, the top ends were not subjected to boundary conditions, allowing them unrestricted movement in all spatial directions. To assess the RC columns’ performance subjected to the compressive load until failure, a concentric static axial load was imposed at the central point of the columns’ top. Instead of the actual load, the load was emulated by applying equivalent displacement. The displacement control method was implemented to prevent costly testing equipment from getting damaged, resulting from the load failure and subsequent material rupture. 25 kN of the concentric load was applied with a 20 mm equivalent displacement. The loading process followed specific parameters as outlined in Table 4.

## Validation of modeling

### Calibration parameters

To properly examine how various model's characteristics and factors affect the model, it is crucial to calibrate and validate the modeling. To achieve this point, a control specimen (50-E75) from the experimental research works^51–53^ was utilized. The impacts of different factors, such as dilation angle (*d*), mesh element types, mesh size, shape factor (${K}_{c}$), and viscosity parameter (*μ*) were assessed by constructing a total of 12 models. These models encompassed various combinations of the aforementioned properties, as detailed in Table 5.

**Table 5:** Parameters of CDP model.

Description	Used value
Dilation angle (*ψ*)	35° (calibrated)
Eccentricity (*ɛ*)	0.1 (default)
Shape factor (*K*_*c*_)	0.667 (default)
Stress ratio (*σ*_*b*0_/*σ*_*c*0_)	1.16 (default)
Viscosity parameter (*μ*)	0.0003 (calibrated)

**Figure 11:**
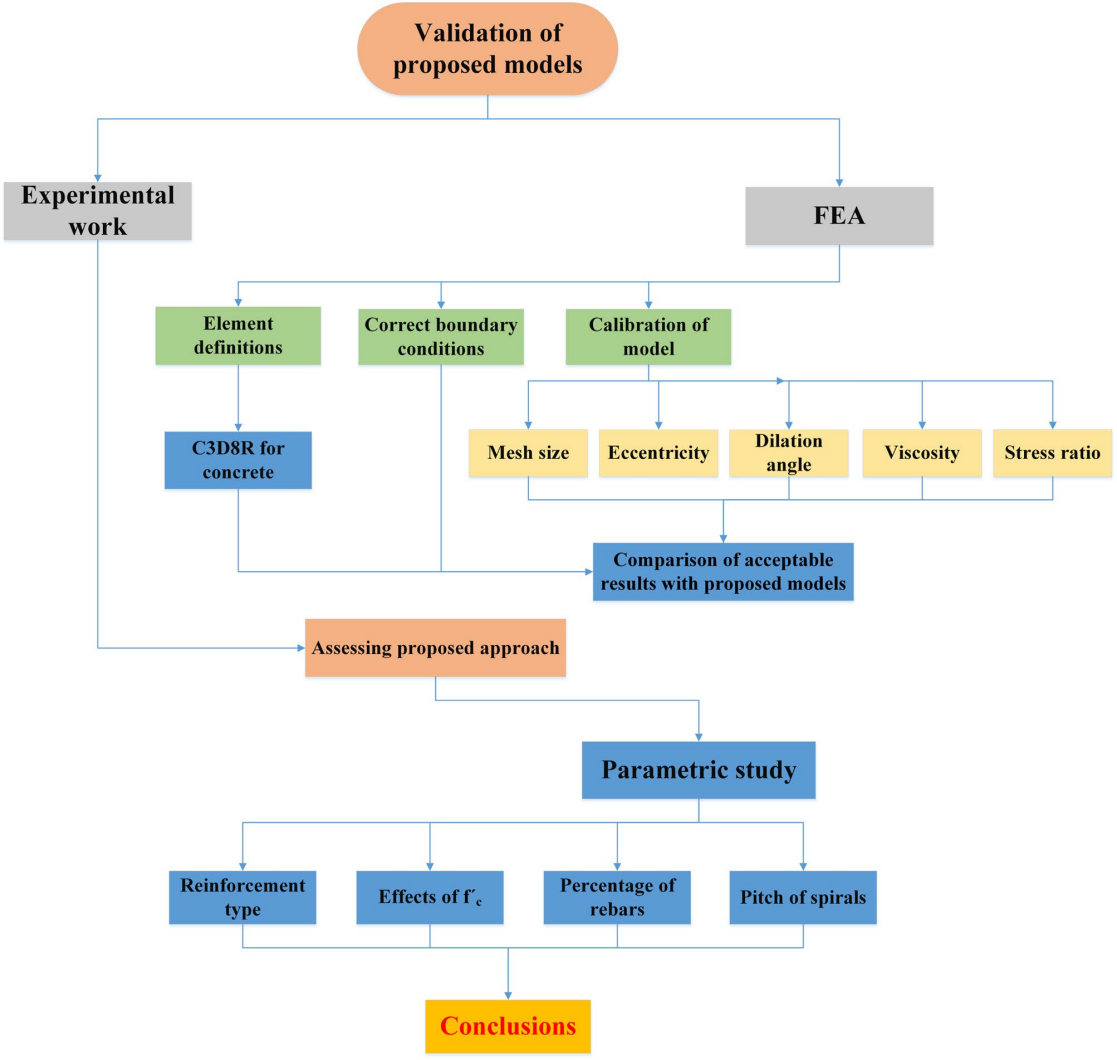
Flow chart of validation and parametric studies.

The control model, which was the validated model, was utilized to conduct FEA and parametric study on each of the simulated RC column specimens. This process is depicted in Fig. 11.

### Viscosity parameter (${\varvec{\mu}}$***)***

**Figure 12:**
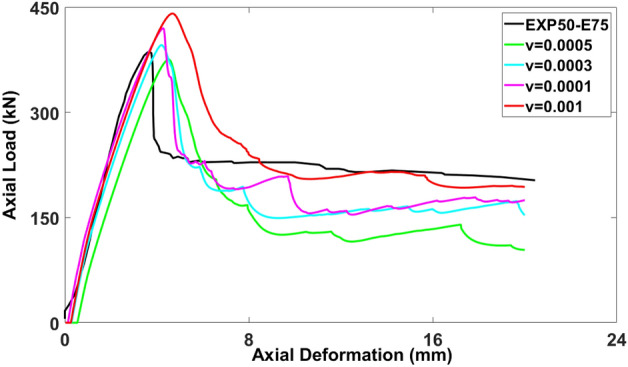
Calibration of viscosity parameter for modeling.

The selection of the viscosity parameter for the model is greatly influenced by the initial and maximum time increments. To approach the desired value as closely as possible, the calibration process began with smaller viscosity values, approximately 15% of the time step increment, as recommended by previous studies^36–38^. The control model was tested with the viscosity values of 0.001, 0.0001, 0.0003, and 0.0005. Figure 12 displays the variation in the load-deformation curves with varying viscosities. Regarding the axial load-carrying capacity, the models with the viscosities of 0.001 (440.82 kN), 0.0001 (419.84 kN), 0.0003 (395.93 kN), and 0.0005 (375.64 kN) exhibited differences of 14.31%, 8.87%, 2.67%, and − 2.65%, respectively, compared to the experimental test result as 385.644 kN. Consequently, the closest match to the experimental result was obtained with the viscosity value of 0.0003. This viscosity value resulted in the model’s behavior closely resembling that of the control model, demonstrating the significance of choosing an appropriate viscosity parameter for accurate simulation.

### Dilation angle (*d*)

**Figure 13:**
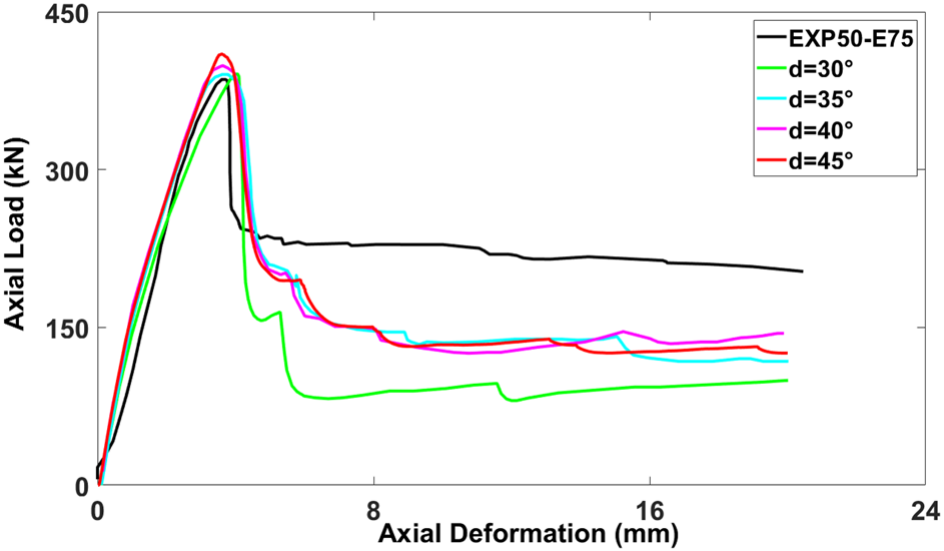
Calibration of dilation angle parameter for modeling.

As presented in Fig. 13, the influence of altering the dilation angle on the load-deformation curves was less considerable compared to the viscosity effect. To achieve the most precise outcome, the dilation angles of 30°, 35°, 40°, and 45° were employed ^36–38^. The axial load-carrying capacity of the models with the dilation angles of 45° (409.87 kN), 40° (398.87 kN), 35° (390.39 kN), and 30° (391.128 kN) exhibited the errors of 6.28%, 3.43%, 1.23%, and 1.42%, respectively, in comparison to the experimental test result (385.644 kN). Thus, the best correspondence was obtained with the dilation angle of 35°.

### Mesh size

**Figure 14:**
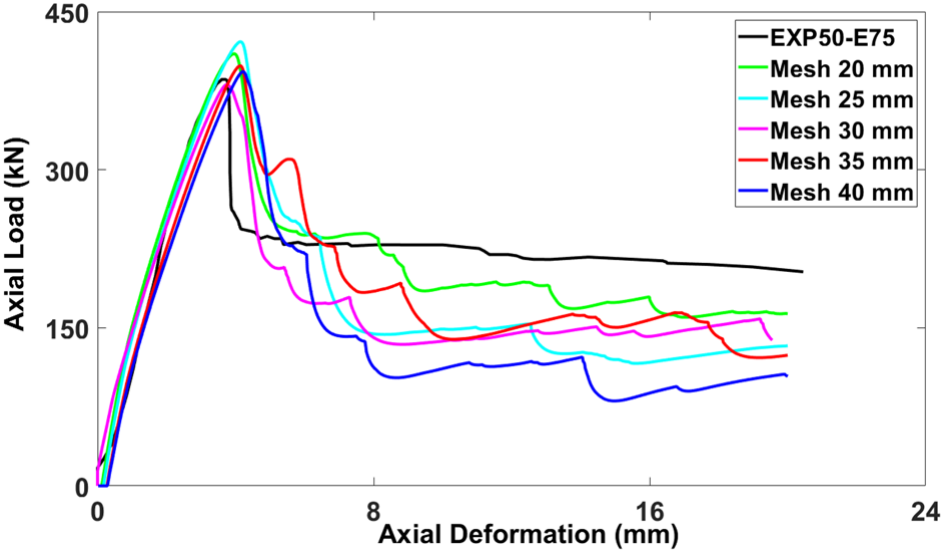
Calibration of mesh size parameter for modeling.

Accurate prediction of numerical results relies heavily on the appropriate selection of mesh size. Opting for a larger mesh size leads to increased discrepancies between the numerical and experimental values, thus compromising the accuracy. The use of different mesh sizes, whether finer or coarser, can trigger localized strain that concentrates strain within a limited selected component and causes the convergence of a numerical model to fail. Appropriate sizes of mesh were employed to ensure a closer alignment between the numerical and experimental results. Figure 14 indicates the axial response of the model for various mesh sizes of 20 mm, 25 mm, 30 mm, 35 mm, and 40 mm^36–38^. The axial load-carrying capacity of the models with the mesh sizes of 20 mm (410.061 kN), 25 mm (421.45 kN), 30 mm (380.93 kN), 35 mm (398.55 kN), and 40 mm (393.12 kN) gave the differences of 6.33%, 9.28%, − 1.22%, 3.35%, and 1.94%, respectively, compared to the experimental test result as 385.644 kN. The analysis of Fig. 14 suggests that the most precise curve was achieved with the mesh size of 40 mm. Furthermore, using smaller sizes of mesh prolonged the analysis time without improving the accuracy significantly.

## Comparison of experimental and numerical results

### Control model (50-E75)

**Figure 15:**
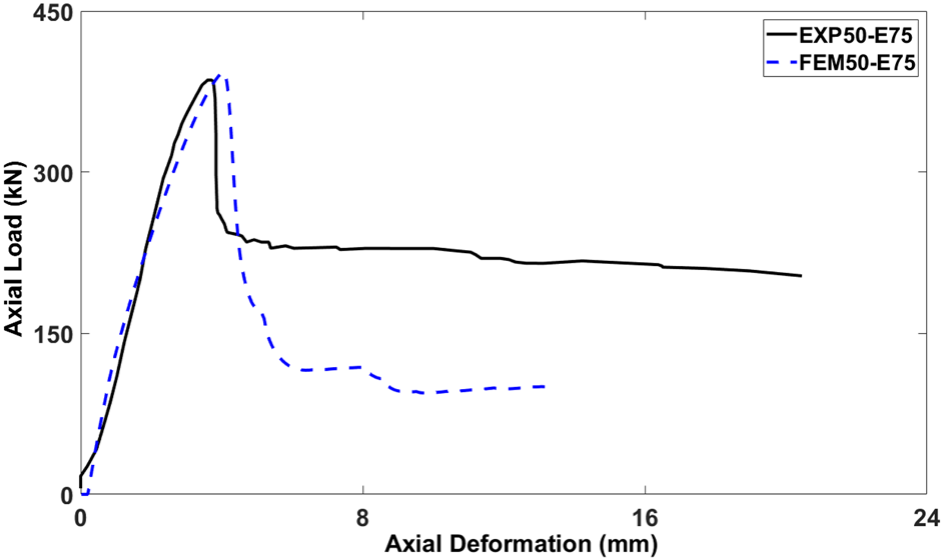
Comparison of experimental (EXP) and FEM results.

Figure 15 presents the control model’s load-deformation curve (50-E75) and the corresponding experimental result. Through the extensive calibration and trial models, the mesh size, viscosity, and dilation angle were refined to their final values of 40 mm, 0.0003, and 35°, respectively. The experimental study carried out by Elchalakani et al.^51–53^ served as the benchmark against which the control model was evaluated. The control model's load-deformation curve closely followed the trajectory of the experimental load-deformation curve, as illustrated in Fig. 15. The FEA results indicated only 3.59% difference in the axial load-carrying capacity and -16.01% difference in the axial deformation compared to the experimental findings. These results demonstrated a close agreement between the experimental and numerical results. Nevertheless, the behavior of the numerical model's post-peak did not accurately match the experimental curve. Because the damage criteria for GFRP bars were not considered, this inconsistency in the post-peak response can be attributed to their linear elastic properties.

### Ultimate axial load-carrying capacity and associated axial deformation

**Figure 16:**
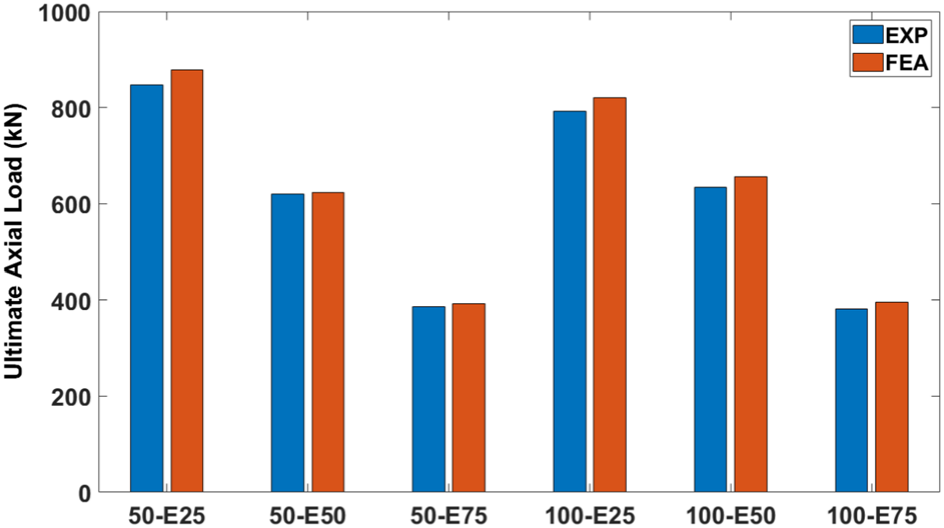
Ultimate axial load-carrying capacities (FEA versus EXP).

**Figure 17:**
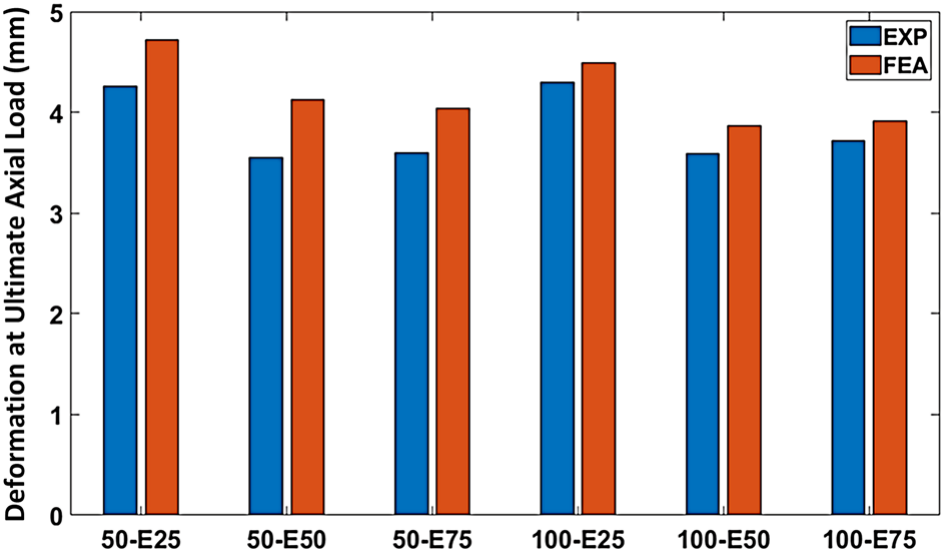
Deformations at ultimate axial load-carrying capacities (FEA versus EXP).

Table 6 provides a comprehensive overview of the ultimate axial load-carrying capacity and associated deformation values obtained from both experimental measurements and FEA for all the columns. Graphical representation of these results can be observed in Figs. 16 and 17. The values of the ultimate axial load-carrying capacity obtained by the modeled columns, namely 50-E25, 50-E50, 50-E75, 100-E25, 100-E50, and 100-E75, along with their respective deformations, are represented in Table 6.

**Table 6:** Comparison of ultimate axial load-carrying capacity and deformation (FEA versus EXP).

No.	Specimen	Ultimate axial load-carrying capacity (kN)			Deformation (mm)		
		EXP	FEA	Difference (%)	EXP	FEA	Difference (%)
1	50-E25	847.53	877.95	3.59	4.26	4.72	10.80
2	50-E50	619.43	623.54	0.66	3.56	4.13	16..01
3	50-E75	385.64	393.08	1.93	3.60	4.04	12.22
4	100-E25	792.12	820.73	3.61	4.30	4.50	4.65
5	100-E50	633.70	655.92	3.51	3.59	3.87	7.8
6	100-E75	381.91	395.28	3.5	3.72	3.92	5.38

The ultimate axial load-carrying capacity of the modeled columns (50-E25, 50-E50, and 50-E75) compared to the EXP specimens were overestimated by 3.59%, 0.66%, and 1.93%, respectively. Similarly, the ultimate axial load-carrying capacity of 100-E25, 100-E50, and 100-E75 were overestimated by 3.61%, 3.51%, and 3.5%, respectively.

Comparisons of the obtained numerical values for the ultimate axial load-carrying capacity and deformation of the columns with different stirrups spacing and eccentricity values are listed in Table 7. The percentage of the reduction in the ultimate axial load-carrying capacity between 50-E25 and 100-E25 was 6.52%, which was quite expected due to the increase in the stirrups spacing. However, the ultimate axial load-carrying capacity enhanced by 5.19% between 50-E50 and 100-E50 and by 0.56 between 50-E75 and 100-E75.

**Table 7:** Comparison of numerical values of ultimate axial load-carrying capacity and deformation for different stirrups spacing and eccentricity values.

Specimen	Ultimate axial load-carrying capacity		Deformation	
	Value (kN)	Difference (%)	Value (mm)	Difference (%)
50-E25	877.95	6.52	4.72	4.66
100-E25	820.73		4.5	
50-E50	623.54	− 5.19	4.13	6.3
100-E50	655.92		3.87	
50-E75	393.08	− 0.56	4.04	2.97
100-E75	395.28		3.92	

### Behavior with respect to axial loads and axial deformations

**Figure 18:**
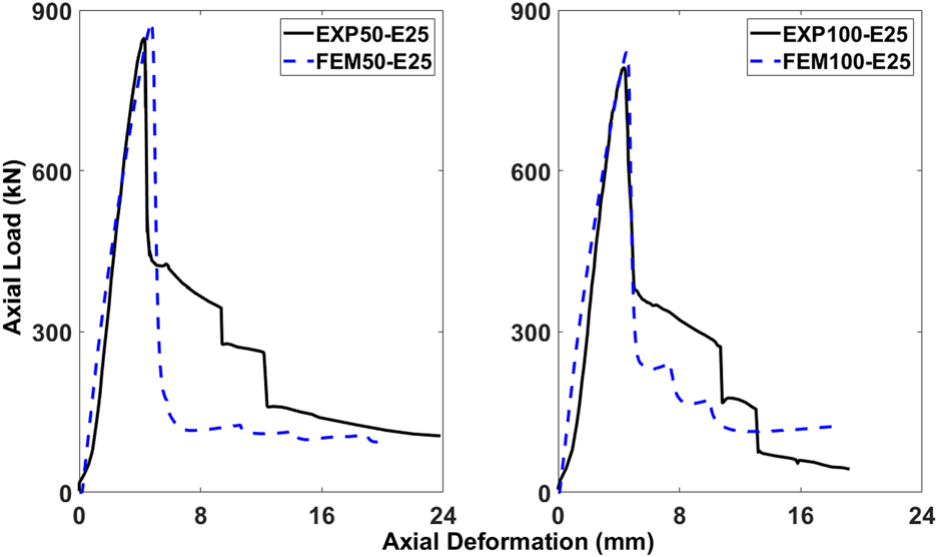
Comparison of FEM and EXP results for columns having 25 mm eccentricity with different spacing of stirrups.

**Figure 19:**
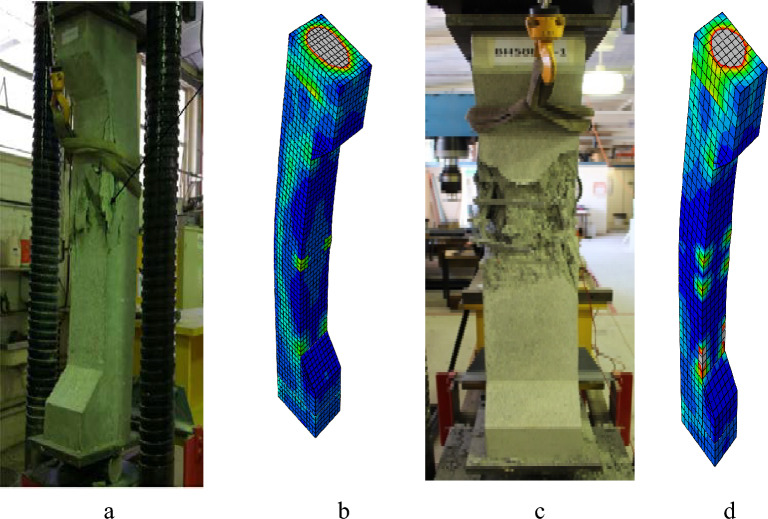
Failure modes of columns: (**a**) EXP50-E25, (**b**) FEM50-E25, (**c**) EXP100-E25, (**d**) FEM100-E25.

**Figure 20:**
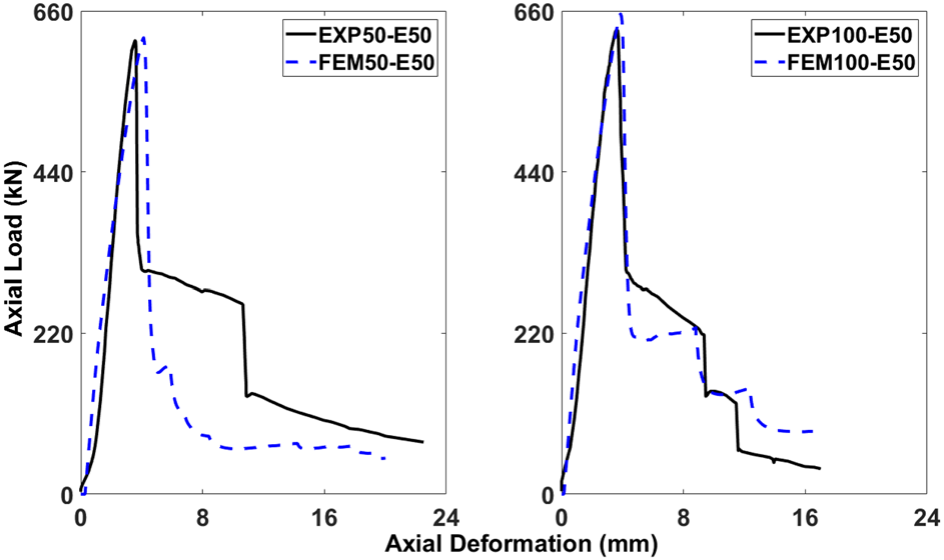
Comparison of FEM and EXP results for columns having 50 mm eccentricity with different spacing of stirrups.

**Figure 21:**
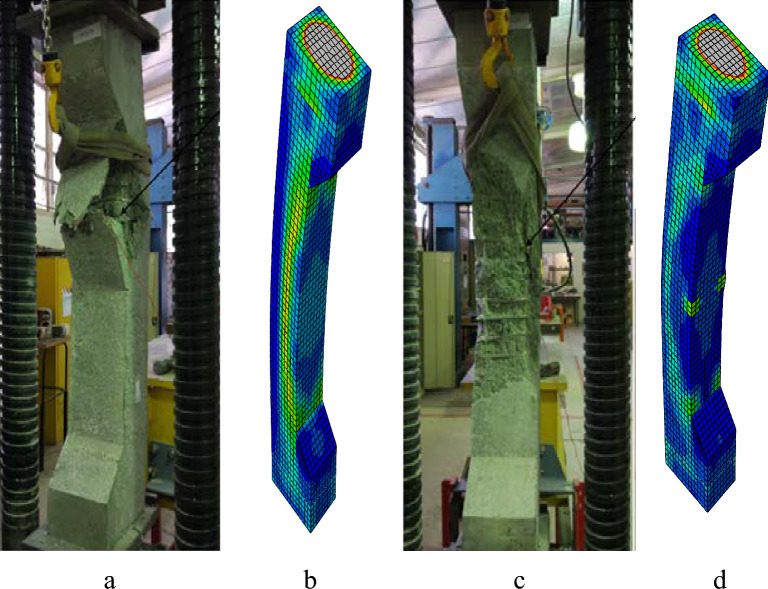
Failure modes of columns: (**a**) EXP50-E50, (**b**) FEM50-E50, (**c**) EXP100-E50, (**d**) FEM100-E50.

**Figure 22:**
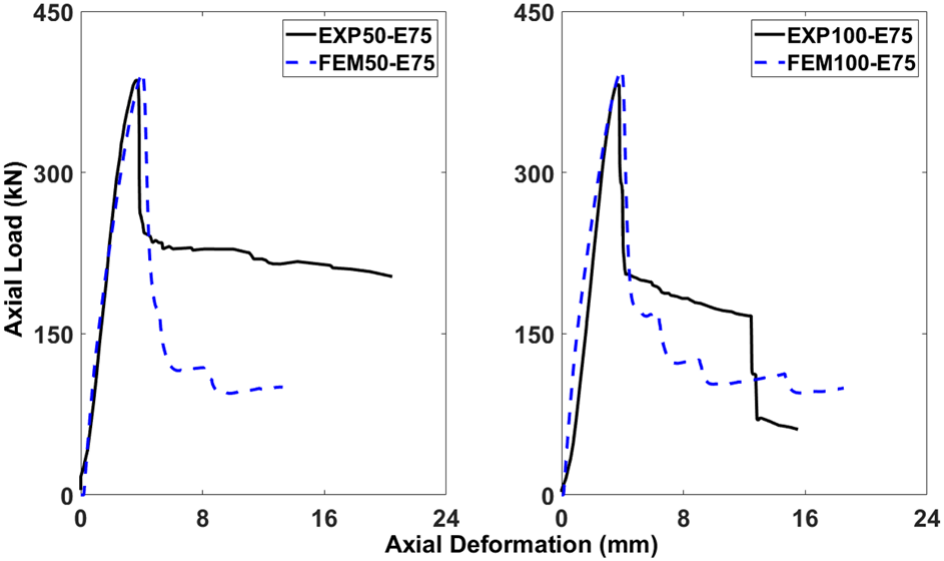
Comparison of FEM and EXP results for columns having 75 mm eccentricity with different spacing of stirrups.

**Figure 23:**
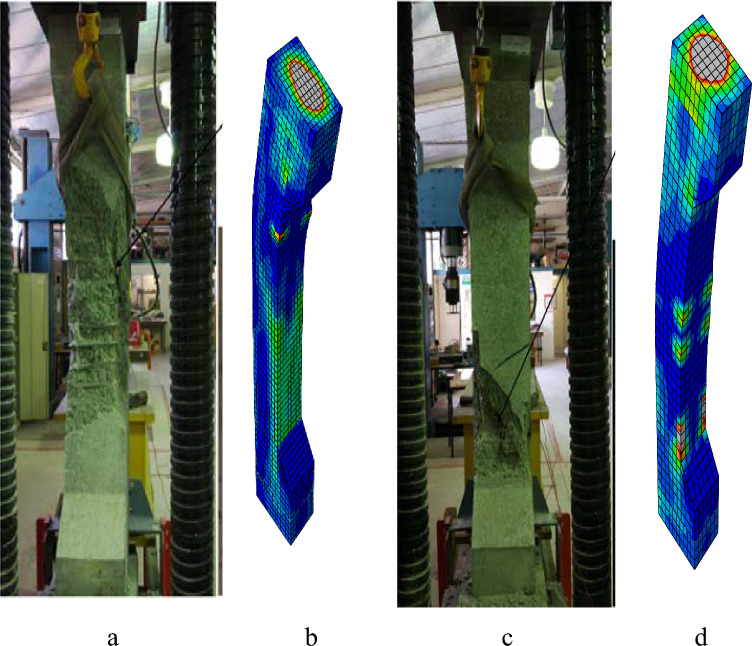
Failure modes of columns: (**a**) EXP50-E75, (**b**) FEM50-E75, (**c**) EXP100-E75, (**d**) FEM100-E75.

Figures 18, 19, 20, 21, 22, and 23 present comparisons between the results obtained from FEM and EXP. After reaching the peak load, there is a decrease in the load-carrying capacity (Figs. 18, 20, and 22) due to concrete cover spalling. However, a subsequent rise in the load-carrying capacity was observed, which can be attributed to the constraining impact of the stainless-steel stirrups wrapped around the internal concrete core. Linear elastic curves were witnessed in the models of all columns before reaching the peak load, followed by a second peak caused by the confinement provided by GFRP reinforcements, and finally, failure resulting from the rupture of the reinforcements. Prior to the peak, the numerical models accurately described the load-deformation response of the specimens. Nevertheless, the numerical simulation overestimated the observed values in the post-peak period and continued to do so until failure, deviating from the experimental data. The damaging criterion for GFRP reinforcements was not specified because they were modeled as linear elastic materials, which is why there was a difference in the post-peak phase.

Figures 19, 21, and 23 show the comparisons of the failure modes of the finite element models and experimental specimens. The equivalent plastic stress’s (PEEQ)^49^ orientation is perpendicular to the way concrete cracks spread. The behavior of cracks and failure modes in the examined columns was therefore analyzed using PEEQ models created for these columns. In order to see and understand how the axial strain formed at the locations where fractures appeared, all column strain models were also investigated. PEEQ is frequently employed to assess a system’s plastic behavior, including the degree of the deformation that can occur prior to failure. PEEQ indicates the irreversible strain in the material associated with the plastic deformation. The contour plots in Figs. 19, 21, and 23 depict the maximum value of concrete strain in percentage. The PEEQ model and strain model for each column provide insights into the development of the axial strains and stresses at the locations where the concrete cover experienced spalling. This indicated that the proposed finite element model could reasonably anticipate and elucidate the progression of cracks and columns’ failure patterns.

#### Columns under eccentric loading of 25 mm

The load-deformation behavior of 50-E25 and 100-E25 started out linearly before entering a brief nonlinear phase just prior to the peak load. The load-deformation curve’s nonlinear phase represented the beginning and growth of small cracks in the outer concrete core, which aligned with the observations made during the experimental work. The percentage differences between the FEM and EXP results for 50-E25 were 3.59% for the ultimate axial load-carrying capacity and 10.79% for the peak deformation, both falling within the acceptable limits. Similarly, for 100-E25, the percentage differences between the FEM and EXP results were 3.61% for the ultimate axial load-carrying capacity and 4.63% for the peak deformation (Fig. 18).

In Fig. 19, the breakage of concrete and the deformation of stirrups are evident in 50-E25 and 100-E25, leading to the subsequent longitudinal bars buckling, ultimately failing to succeed. Nevertheless, the modeling exhibited a similar pattern, with a higher concentration of stress in the center of 50-E25 and 100-E25, attributed to the failure of both reinforcements.

#### Columns under eccentric loading of 50 mm

As displayed in Fig. 20, 50-E50 and 100-E50 initially demonstrated a linear load-deformation response, and then there was a nonlinear phase prior to reaching the peak load. The emergence and progression of tiny cracks in the exterior concrete core were indicated by the load-deformation curve’s nonlinearity, which aligned with the findings from the experimental work. The percentage differences between the FEM and EXP results for 50-E50 were 0.66% and 16.16% for the ultimate axial load-carrying capacity and peak deformation, respectively, which are within the acceptable limits. Also, the percentage differences between the FEM and EXP results for 100-E50 were 3.51% and 7.66% for the ultimate axial load-carrying capacity and peak deformation, respectively (Fig. 20).

Figure 21 illustrates the occurrence of concrete fracture and the deformation of stirrups in 50-E50 and 100-E50, leading to the subsequent longitudinal bar buckling and eventual failure. However, the modeling revealed a similar outcome, with a higher concentration of stress in the central region of 50-E50 and 100-E50, which was related to the failure of both reinforcements.

#### Columns under eccentric loading of 75 mm

According to Fig. 22, 50-E75 and 100-E75 exhibited a linear load-deformation trend, as the load reached its peak, a nonlinear phase quickly followed. The load-deformation curve indicated that cracks were initiating and propagating in the outer concrete core, consistent with the observations made during the experimental study. The percentage differences between the FEM and EXP results for 50-E75 were 1.93% and 12.06% for the ultimate axial load-carrying capacity and peak deformation, respectively, which were within the acceptable limits. Moreover, the percentage differences bewteen the FEM and EXP results for 100-E75 were 3.5% and 5.43% for the ultimate axial load-carrying capacity and peak deformation, respectively (Fig. 22).

The fracture of concrete and opening of the stirrups are evident in Fig. 23 for 50-E75 and 100-E75, then sudden buckling of the longitudinal bars occurred, which led to the failure. Nevertheless, the same was provided by the modeling, with greater stress concentration for 50-E75 and 100-E75 in the middle region owing to the failure of both reinforcements.

### Ductility of columns

The property of being able to endure the plastic deformation without failing is referred to as ductility^54^. It represents the ability of a structural element to withstand applied forces even after reaching the limit of the compressive strength. Ductility can be assigned to various mechanical parameters, such as energy, axial strain, rotation, or deformation associated with the element. Comparing and contrasting the ductility of identical-sized hollow RC columns reinforced with GFRP and steel was done. Equation (7) was used to obtain the ductility factor (*DF*) for the columns of this study.

7 $$DF= \frac{{A}_{\Delta 85}}{{A}_{\Delta 75}}$$

**Figure 24:**
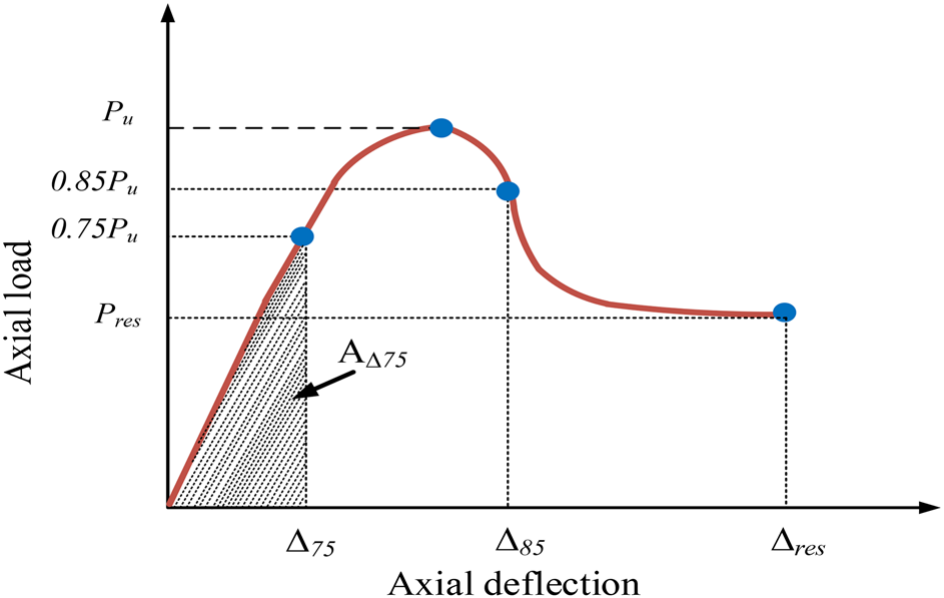
DF.

The graphical representation of ${A}_{\Delta 75}$ and ${A}_{\Delta 85}$ can be observed in Fig. 24. ${A}_{\Delta 75}$ denotes the region of the curve that is below a certain point (${\Delta }_{75}$), where the axial distortion inside the elastic area reaches 75% of the maximum compressive strength. Conversely, ${A}_{\Delta 85}$ signifies the integral of the curve until a specific point (${\Delta }_{85}$), when the axial deflection in the inelastic phase attains 85% of the ultimate compressive strength. By utilizing the method based on the area under the curve, DF for all experimental and numerical models was determined to be 1.30. It should be noted that all columns possessed similar geometry and reinforcement, with the only variation being in the stirrups spacing and eccentricity values, as summarized in Table 8.

**Table 8:** DFs of experimental and numerical values.

Specimen	*P*_*u* (kN)_	Peak deflection (mm)	0.75*P*_*u* (kN)_	0.75 peak deflection (mm)	*A*_Δ75_	0.85*P*_*u* (kN)_	0.85 peak deflection (mm)	*A*_Δ85_	DF
EXP50-E25	847.53	4.26	635.65	3.20	1015.46	720.40	3.62	1322.36	1.30
FEM50-E25	877.95	4.72	658.46	3.54	1165.46	746.26	4.01	1517.68	1.30
EXP50-E50	619.43	3.56	464.57	2.67	619.40	526.51	3.02	806.59	1.30
FEM50-E50	623.54	4.13	467.65	3.10	724.28	530.01	3.51	943.17	1.30
EXP50-E75	385.64	3.60	289.23	2.70	390.78	327.80	3.06	508.88	1.30
FEM50-E75	393.08	4.04	294.81	3.03	446.35	334.12	3.43	581.25	1.30
EXP100-E25	792.12	4.30	594.09	3.22	957.64	673.31	3.65	1247.06	1.30
FEM100-E25	820.73	4.50	615.55	3.37	1038.12	697.62	3.82	1351.86	1.30
EXP100-E50	633.70	3.59	475.27	2.69	640.31	538.64	3.05	833.82	1.30
FEM100-E50	655.92	3.87	491.94	2.90	713.52	557.53	3.29	929.16	1.30
EXP100-E75	381.91	3.72	286.43	2.79	399.07	324.62	3.16	519.68	1.30
FEM100-E75	395.28	3.92	296.46	2.94	435.46	335.99	3.33	567.06	1.30

### Summary of validated model

The findings related to the calibrated finite element models revealed that the control model's load-deformation curve (50-E75) was in a close agreement with the experimental result, as represented by Fig. 15. The peak loads of the columns deviated on average by 1.93% between the experimental and numerical curves. Additionally, in the visualization of cracks within the column, the strain model and PEEQ model displayed the stress and axial strain at the precise locations of real cracks. These findings collectively demonstrated the suitability of the proposed finite element model for conducting subsequent parametric investigations.

## Conclusions

The goal of this study was to look at how GFRP columns respond axially subjected to eccentric loading using the ABAQUS software. FEA involved modeling the RC columns with the CPD model and representing reinforcements as linear elastic materials. The main findings are summarized as follows:

- The findings from the numerical analysis revealed that specific input parameters had an impact on the ultimate axial load-carrying capacity of GFRP-RC columns. The most critical parameters were the mesh size and viscosity parameter. Other parameters affecting the ultimate axial load-carrying capacity of the columns were the shape factor, dilation angle, and mesh type. Thus, any finite element model should be calibrated for these variables before using it for a numerical study.
- The numerical models calibrated with experimental data demonstrated comparable results to the experimental findings. The load-deformation responses of all the columns' finite element models closely matched the experimental reponses, particularly in the pre-peak phase. This calibration process was a critical step to ensure the validity and accuracy of the finite element model when predicting the behavior under new loading scenarios.
- ABAQUS could effectively simulate the post-peak response of the load-deformation curves when GFRP bars and stirrups were utilized. Specifically, the ultimate failure of GFRP reinforcements could not be predicted by the curves. This was probably due to the fact that the damage parameters of GFRP were not as defined as those of concrete. Therefore, further study is required to define the damage pattern of GFRP reinforcements.
- The average variation in the maximum axial load-carrying capacity of the experimental results compared to the FEA values, was noted to be only 2.8%, while the average difference in their corresponding deformations was -8.21%. This nominal difference between the results uncovered that the numerical model was suitable for implementation in further study of GFRP columns against eccentric loading.
- The numerical investigation was validated against the experimental findings concerning the varied values of load eccentricities and different spacing of stirrups in the GFRP columns.
- Equivalent principal plastic strains were used to visualize the pattern of cracking in the columns. The fracture growth behavior reached was satisfactorily compared using the FEA and actual experimental cracks. This supported the claim that FEA of structural members may be carried out employing ABAQUS without the need for expensive experimental research.
- One area that requires further attention in this research project is the validation of the results obtained from the detailed experimental scheme used for the parametric studies. This work's limitation lies in the need for future research to address this validation aspect.

## Recommendations

The ABAQUS models can be utilized to estimate the load-carrying capacity of the GFRP columns by incorporating specific parameters such as the viscosity parameter, shape factor, dilation angle, and mesh size values of 0.0003, 0.667, 35°, and 40 mm, respectively. The outcomes indicated that the proposed finite element models closely approximated the load-deformation behavior of the tested specimens when compared to their corresponding experimental results.

## Data Availability

All data generated or analyzed during this study are included in the published article.
